# Cricket (*Acheta Domesticus*) Flour as a Novel Ingredient in Hybrid Meatloaves: Effects on Quality Attributes During Storage

**DOI:** 10.1111/1750-3841.70672

**Published:** 2025-11-23

**Authors:** Camila Cristina Avelar de Sousa, Thiago da Matta Pires Cruz, Juliana Sant'Ana Falcão Leite, Adriel da Silva Luz, Gabriela Santana dos Anjos, Ana M. Herrero, Maurício Costa Alves da Silva, Claudia Ruiz‐Capillas, Carlos Pasqualin Cavalheiro

**Affiliations:** ^1^ Laboratório de Inspeção e Tecnologia de Carnes e Derivados (LabCarne) Universidade Federal da Bahia Salvador Bahia Brazil; ^2^ Department of Meat and Fish Products, Institute of Food Science, Technology and Nutrition (ICTAN‐CSIC) INDMEAT group Madrid Spain

**Keywords:** house cricket, meat replacement, microbiological quality, physicochemical properties, quality attributes, storage stability

## Abstract

The rising demand for animal protein challenges the agricultural sector, and edible insects provide a sustainable alternative. This study investigated the impact of replacing lean beef with cricket (*Acheta domesticus*) flour on the physicochemical and microbiological characteristics of hybrid meatloaf during refrigerated storage. Four formulations containing 0%, 7.5%, 10%, and 12.5% cricket flour were tested. Higher levels of cricket flour enhanced protein content, improved the mineral profile, and reduced cooking loss. The pH was not affected by the cricket flour addition; however, the storage period decreased the pH of hybrid meatloaves (*p* < 0.05). Color changes were noted, with hybrid meatloaves becoming darker and browner. Hardness values were initially higher but decreased over storage in hybrid meatloaves, while the control (CM0) exhibited the opposite trend. Storage had a more pronounced effect on lipid oxidation in hybrid meatloaves. However, these treatments exhibited microbiological stability despite having high initial microbial counts. Replacing up to 12.5% of lean beef with cricket flour in hybrid meatloaves was achieved.

## 1 Introduction

Beef is a highly nutritious protein source, providing a balanced amino acid profile and essential micronutrients such as iron, zinc, selenium, vitamin B12, and vitamin D (Rocchetti et al. 2022). However, the rising demand for animal‐based proteins presents a substantial challenge to agriculture, with projected annual meat production expected to rise by up to 200 million tons by 2050 (Alexandratos and Bruinsma 2012). Traditional livestock farming requires significant resources, including water and land (Yang et al. 2021), emphasizing the need for sustainable protein alternatives to meet global food demands (Mariutti et al. 2021).

In this context, edible insects have emerged as a promising alternative protein source, offering high levels of vitamins, lipids, and minerals (Anzani et al. 2020) while being more sustainable than conventional livestock due to their lower greenhouse gas emissions, superior feed conversion efficiency, and reduced water and land usage (Halloran et al. 2016). Despite these advantages, cultural resistance remains a significant barrier, particularly in Western societies, where entomophagy is mainly unfamiliar (Mishyna et al. 2020). Studies indicate that edible insects are less preferred than other alternative proteins, such as plant‐based proteins, cultured meat, and algae (Onwezen et al. 2021). This resistance is primarily driven by psychological factors, including disgust, fear, and neophobia (Erhard et al. 2022), which are exacerbated when insects are visibly present in food. To overcome these challenges, strategies such as incorporating insects in less recognizable forms within familiar food products and ensuring the sensory appeal of insect‐based products have been suggested to improve consumer acceptance (van Huis and Rumpold 2023). Moreover, formulation and serving approaches have also been identified as relevant factors to enhance the overall acceptability of food products containing insect flour (Ho et al. 2022a).

Edible insects have already been successfully integrated into various baked goods, including pasta (Pasini et al. 2022; Ho et al. 2022b), bread (Pyo et al. 2024), and biscuits (Xie et al. 2022), to enhance their protein content. Their nutritional and functional properties also make them suitable for reformulating meat products (Cavalheiro et al. 2023). Notable examples include the incorporation of *Tenebrio molitor*, *Gryllus assimilis*, and *Locusta migratoria* into products such as emulsified cooked sausages (Kim et al. 2016; Choi et al. 2017), meat emulsions (Singh et al. 2023), and beef patties (Gomes Martins et al. 2024).

The European Commission recently authorized the commercialization of frozen, dried, and powdered *Acheta domesticus* (house cricket) as a novel food (European Union 2022). House crickets are particularly promising due to their ease of cultivation, high levels of protein, lipids, and essential minerals, as well as favorable profiles of monounsaturated and polyunsaturated fatty acids (Psarianos et al. 2022; Cavalheiro et al. 2023). Additionally, they have a mild flavor (van Huis 2020), which may enhance consumer acceptance.

Given these considerations, reformulating meat products with cricket warrants further investigation. Some studies have already explored the partial replacement of meat with cricket flour. For instance, Cavalheiro et al. (2023) demonstrated that incorporating 5% cricket flour as a pork replacer enhanced the nutritional profile of frankfurters without compromising their sensory or structural qualities. Other studies have examined the application of cricket flour in cooked sausages (Kim et al. 2017; Ho et al. 2022b), pâtés (Walkowiak et al. 2019), and beef patties (Cavalheiro et al. 2024). However, its use in other meat products remains underexplored. Therefore, evaluating its incorporation at varying levels into widely consumed items, such as meatloaf, which is a restructured meat product renowned for its nutritional value, affordability, and versatility, could contribute to diversifying available options and promoting the acceptance of this sustainable protein source.

However, incorporating cricket flour into meatloaf formulations requires careful consideration, as introducing a new protein source can significantly influence quality attributes. Therefore, this study aimed to evaluate how different levels of beef replacement with cricket flour (0%, 7.5%, 10.0%, and 12.5%) affect the physicochemical, microbiological, and technological quality of hybrid meatloaves during refrigerated storage (7 days at 4°C).

## 2 Materials and Methods

### 2.1 Raw Materials and Meatloaf Manufacturing

Fresh beef knuckle (*M. rectus femoris, M. vastus intermedius, vastus lateralis, vastus medialis*), curing salt (Conatril, Rio Claro, Brazil), sodium chloride (NaCl) (Lebre, Areia Branca, Brazil), garlic powder (Empório Metas, Diadema, Brazil), nutmeg (Tropicana, Lauro de Freitas, Brazil), and sodium erythorbate (SuplementoFácil, São Paulo, Brazil) were obtained from a local market in Salvador, Brazil. Cricket flour, previously characterized by Cavalheiro et al. (2023), was sourced from the UK market (Derby, UK).

Four meatloaf formulations were prepared in duplicate at a private pilot plant and repeated over two days, according to Ribeiro et al. (2023), with slight modifications. Optimal cricket flour levels were determined in preliminary lab tests. A control (CM0) contained beef lean meat (75.0%), water/ice (22.0%), sodium chloride (1.0%), sodium nitrite (0.17%), garlic powder (0.15%), nutmeg powder (0.10%), and sodium erythorbate (0.05%). Hybrid treatments replaced portions of beef with cricket flour: 7.5% (CM75), 10.0% (CM100), and 12.5% (CM125).

The beef was ground through an 8 mm disc in a meat mincer (MCR08 3.0, Arbel, São José do Rio Preto, Brazil), then homogenized with non‐meat ingredients in a mixer (RI7630, Phillips Walita, São Paulo, Brazil) for 3 min on low speed, keeping the final temperature under 10°C. The mixture was distributed into disposable aluminum bowls (WYDA, Brazil) and cooked in an electric oven (Model PFE42P, Philco, Brazil) at 180°C for 20 min, reaching an internal temperature of 80°C. Samples were cooled to 25°C, placed in high‐density polyethylene containers (10.5 cm × 10.5 cm × 5.0 cm), and stored at 4°C for further analysis at days 0 and 7.

### 2.2 Physicochemical Properties

#### 2.2.1 Proximate Composition

Moisture and ash were assessed using AOAC (2005) methods. Protein was determined using a LECO FP‐200 Nitrogen Determinator (Leco Corp., St Joseph, MI, USA), and lipid content was determined using the Bligh and Dyer (1959) method. All determinations were performed in triplicate.

#### 2.2.2 Mineral Content

Freeze‐dried samples (Lyophilized Telstar Cryodos Equipment, Spain) were acid‐digested with nitric acid in a microwave system (ETHOS 1, Milestone. Srl, Sorisole, Italy), as reported previously (Sánchez‐Faure et al. 2020). Sodium (Na), Potassium (K), Phosphorus (P), Magnesium (Mg), Calcium (Ca), Iron (Fe), Zinc (Zn), and Copper (Cu) were quantified on a ContrAA 700 High‐Resolution Continuum Source spectrophotometer (Analytik Jena AG, Jena, Germany) equipped with a xenon short‐arc lamp (GLE, Berlin, Germany). Results were expressed as mg/100 g, with each sample analyzed in triplicate.

#### 2.2.3 Processing Losses

The weight difference between raw and cooked samples was defined as cooking loss (%). In addition, the weight difference of meatloaves after seven days of storage at 4°C was defined as cooling loss (%).

#### 2.2.4 pH

The pH was determined using a digital pH meter (model Mpa‐210; Tecnopon, São Paulo, Brazil) calibrated with standard buffers at pH 4.00 and 7.00 (25 ± 1°C). Three readings were taken for each treatment using 10 g of each sample homogenized in 90 mL of distilled water.

#### 2.2.5 Instrumental Color

Color parameters were measured in triplicate using a Chroma Meter CR‐5 (Minolta Business Technologies Inc., Tokyo, Japan), with illuminant D‐65, 10 standard observer, and 8 mm aperture. Meatloaves were removed from the packaging and exposed to atmospheric air for 10 min before color measurement. The instrumental color CIELAB (lightness—L*, redness—a*, yellowness—b*) was measured from the surface of meatloaves. The total color difference (ΔE*) relative to the control (CM0) was calculated as follows:

$$\Delta E^{*}=\sqrt{{\left(L-L_{0}\right)}^{2}+{\left(a-a_{0}\right)}^{2}+{\left(b-b_{0}\right)}^{2}}$$

#### 2.2.6 Texture Profile

Texture profile analysis (TPA) was conducted on a TA.XT Express analyzer (Stable Micro Systems Ltd., Surrey, England), evaluating hardness (*N*), chewiness (*N* × mm), springiness (mm), and cohesiveness. Meatloaf samples were cut into cubes measuring 1.0 cm × 1.0 cm × 1.0 cm for analysis. The TPA used a P/36R probe with these parameters: pre‐test speed of 2 mm/s, test speed of 1 mm/s, post‐test speed of 10 mm/s; trigger force of 5.0 g, and compression distance of 5 mm (Yang et al. 2021).

#### 2.2.7 Lipid Oxidation

Lipid oxidation was determined using the TBARS assay, following Triki et al. (2013) with minor modifications. Five grams of each sample was homogenized in 35 mL of 7.5% trichloroacetic acid (Êxodo Científica, São Paulo, Brazil) for 30 s in a stomacher (Logen 1901, Logen Scientific, São Paulo, Brazil), filtered, and mixed with 0.02 M thiobarbituric acid (Êxodo Científica, São Paulo, Brazil). After 20 h in the dark at 20 ± 1.5°C, color was read at 532 nm on a spectrophotometer (SP‐22, Biospectro, Curitiba, Brazil). Results are expressed as mg MDA/kg based on a calibration curve of 1, 1, 3, 3‐tetraethoxypropane. Nine measurements were performed per treatment.

### 2.3 Microbiological Properties

A 10 g sample was aseptically homogenized in 90 mL of 0.1% peptone water (Kasvi, São José dos Pinhais, Brazil) for 30 s. Serial dilutions were plated on different media for microbial counts: plate count agar (BD Difco, Le Pont de Claix, France) was used for mesophilic count (48 h at 35°C) and psychrophilic aerobic counts (seven days at 4°C); violet red bile agar (Kasvi) for enumeration of *Enterobacteriaceae* (48 h at 35°C); Baird‐Parker agar (Kasvi) for coagulase‐positive staphylococci count (incubation for 48 h at 35°C); MRS agar (Acumedia, Michigan, USA) for lactic acid bacteria counts (48 h at 35°C). *Salmonella* presence was also tested. Counts are reported as log CFU/g, with duplicate determinations.

### 2.4 Statistical Analysis

The experimental design was completely randomized, with two independent batches of each treatment, processed and analyzed separately. A generalized linear model was applied to evaluate the results using SPSS 25.0 (SPSS Inc., Chicago, IL, USA). Fixed effects included treatments and storage days, and replicates were treated as a random effect. The interaction between fixed effects was evaluated. Samples comparisons were made using ANOVA with Tukey's test at the 5% level was used to compare the means.

## 3 Results and Discussion

### 3.1 Physicochemical Properties

#### 3.1.1 Proximate Composition

The incorporation of cricket flour significantly influenced (*p* < 0.05) the moisture, protein, and fat contents of meatloaves (Table 1). CM0 exhibited a moisture content of 67.13%, whereas the hybrid samples showed lower values. Conversely, protein content increased significantly (*p* < 0.05) in hybrid treatments, reaching values between 28.02% and 29.20% compared to 22.56% in CM0. Similarly, fat content rose significantly (*p* < 0.05), from 4.15% to 5.11% in the hybrid meatloaves, compared to 2.38% in CM0. As previously reported, these changes in proximate composition are consistent with the cricket flour's protein and fat content (Cavalheiro et al. 2023). Since beef was the primary ingredient in these formulations, the increase in cricket flour corresponded to a higher fat content in the hybrid samples, as no additional lipid sources were included. Ash content remained unchanged across treatments (*p* > 0.05). Similar findings have been reported in other studies, including reduced moisture content, increased protein and fat levels, and stable ash content in meat products with edible insect incorporation (Kim et al. 2017; Cavalheiro et al. 2023).

**TABLE 1:** Proximate composition (%) and mineral content (mg/100 g) of meatloaves with different levels of cricket flour.

—	CM0	CM75	CM100	CM125	SEM	*P*‐value
Proximate composition		—	—	—	—	—
Moisture	67.13^A^	57.24^B^	56.41^C^	54.03^D^	1.89	***
Protein	22.56^D^	28.02^C^	28.70^B^	29.20^A^	1.00	***
Fat	2.38^C^	4.15^B^	4.50^AB^	5.10^A^	0.31	***
Ash	4.38	4.39	4.40	4.32	0.08	n.s.
Mineral content		—	—	—	—	—
Na	1654.81^AB^	1759.68^A^	1533.09^B^	1576.03^AB^	34.76	*
K	403.75	502.03	492.99	533.45	20.81	n.s.
P	158.04^B^	203.33^A^	186.11^AB^	202.97^A^	7.54	*
Mg	27.19^B^	34.84^A^	34.43^A^	35.40^A^	1.28	***
Ca	12.16^C^	26.02^B^	25.06^B^	33.58^A^	2.92	***
Fe	8.00^A^	3.93^B^	2.83^C^	4.62^B^	0.73	***
Zn	6.75^AB^	6.46^B^	6.44^B^	7.36^A^	0.07	*
Cu	0.11^C^	0.34^B^	0.35^AB^	0.47^A^	0.05	***

*Note*: Means with different uppercase superscripts (A–C) in the same row indicate significant differences among treatments (*P* < 0.05). SEM: standard error of the mean. CM0: meatloaf without cricket flour; CM75: meatloaf with 7.5% cricket flour; CM100: meatloaf with 10% cricket flour; CM125: meatloaf with 12.5% cricket flour. t: treatments (CM0, CM75, CM100, CM125); *: *p <* 0.05; **: *p <* 0.01; ***: *p <* 0.001; n.s.: not significant.

#### 3.1.2 Mineral Content

Previous analyses of cricket flour have highlighted its richness in essential micronutrients (Cavalheiro et al. 2023). While ash content remained consistent (*p* > 0.05) across treatments (Table 1), hybrid meatloaves exhibited significant differences in mineral composition compared to CM0. Key minerals included Na, K, and P, with Na levels being exceptionally high due to the addition of NaCl, curing salt, and sodium erythorbate during processing. Sodium content ranged from 1533.09 to 1759.68 mg/100 g (Table 1), with no clear association between Na levels and cricket flour addition, as CM0 and CM125 showed similar values (*p* > 0.05). These findings align with previous studies on cricket flour in emulsified meat products (Kim et al. 2017; Cavalheiro et al. 2023). The relation of Zn content and cricket flour levels was also not observed (Table 1). Cricket flour notably enhanced (*p* < 0.05) the P, Mg, Ca, and Cu contents, likely reflecting the micronutrient profile of cricket flour (Cavalheiro et al. 2023). However, even with the Fe content in cricket flour (5.40 mg/100 g) (Cavalheiro et al. 2023), meatloaves with cricket flour addition showed lower (*p* < 0.05) Fe content than CM0. Nevertheless, CM125 exhibited the highest Fe content among the hybrid formulations and, according to European legislation, can be classified as high in Fe. Moreover, all hybrid meatloaves may be classified as a source of K, P, and Fe, and as high in Zn and Cu (European Union 2006).

Our findings indicate that replacing 7.5% of beef with cricket flour can enhance the mineral profile of hybrid meatloaves, particularly for P, Mg, Ca, and Cu, minerals essential for bone health and metabolic functions. P plays a crucial role in RNA and DNA synthesis, ATP production, and protein synthesis (Udomsil et al. 2019). Notably, the inclusion of cricket flour did not increase Na levels, a significant consideration given the health risks associated with excessive sodium intake (Jaques et al. 2021).

#### 3.1.3 Processing Losses

**FIGURE 1:**
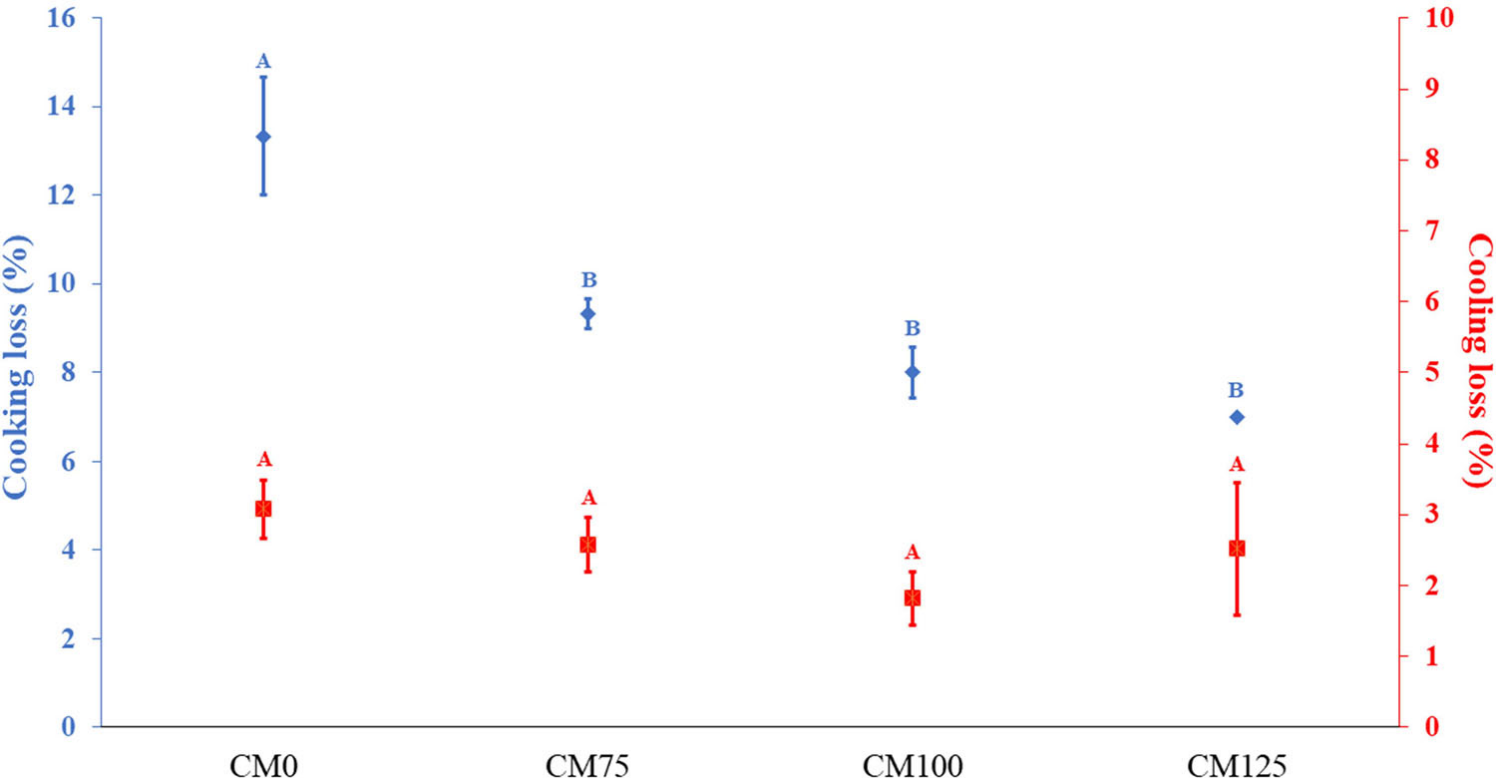
Processing losses (%) of hybrid meatloaves during storage time. Different uppercase superscripts (A–B) indicate significant differences among treatments (*p <* 0.05). Error bars correspond to the SEM. CM0: meatloaf without cricket flour; CM75: meatloaf with 7.5% cricket flour; CM100: meatloaf with 10% cricket flour; CM125: meatloaf with 12.5% cricket flour.

Minimizing processing losses can substantially improve both product yield and economic efficiency. It was observed that the incorporation of cricket flour significantly reduced cooking loss (*p* < 0.05) in hybrid meatloaves, resulting in a higher yield than the CM0 treatment (Figure 1). This can be attributed to the higher protein and lower moisture contents observed in the hybrid treatments (Table 1), consistent with the findings previously reported by Cavalheiro et al. (2023) in frankfurters. The incorporation of cricket flour significantly improves water‐holding and oil‐holding capacities in meat products, which are correlated with reduced cooking loss (Cavalheiro et al. 2024). Besides, Kim et al. (2017) demonstrated that cricket flour also enhances protein solubility and gel formation ability, mechanisms that contribute to lower water and fat exudation during the cooking process. However, increasing cricket flour levels did not further influence cooking loss (*p* > 0.05), nor did its addition significantly affect cooling loss across treatments (*p* > 0.05).

#### 3.1.4 pH

**FIGURE 2:**
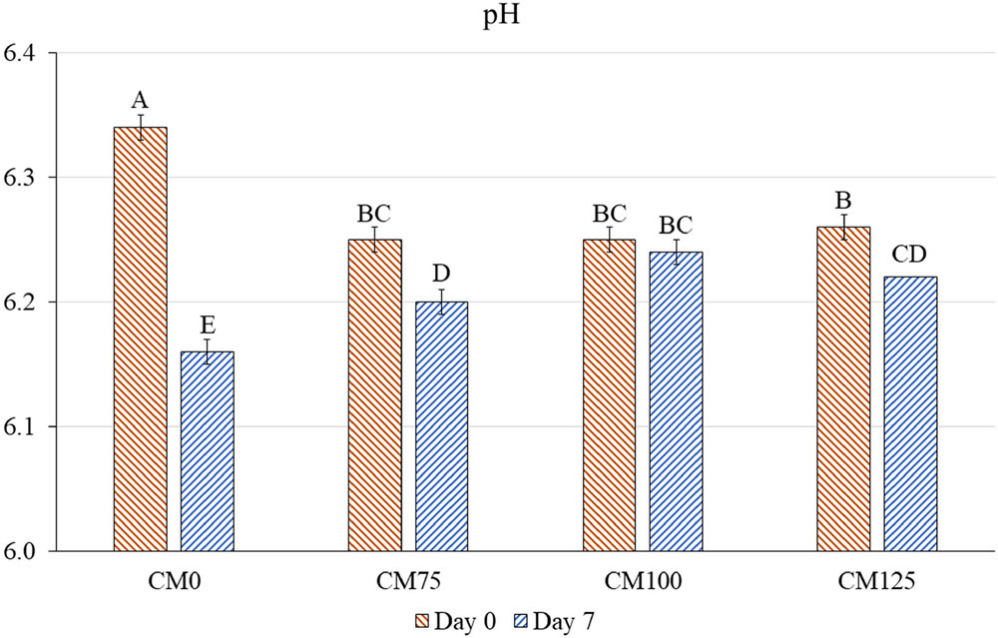
pH of hybrid meatloaves elaborated with cricket flour in different concentrations during storage time. Different uppercase superscripts (A–E) indicate significant differences among treatments (*p <* 0.05). Error bars correspond to the SEM. CM0: meatloaf without cricket flour; CM75: meatloaf with 7.5% cricket flour; CM100: meatloaf with 10% cricket flour; CM125: meatloaf with 12.5% cricket flour.

A significant interaction (*p* < 0.05) was observed between treatments and storage days for the pH values of meatloaves (Figure 2). In this study, CM0 exhibited the highest (*p* < 0.05) pH value (6.34) at day 0, which was higher than those values previously reported for meatloaf (Ribeiro et al. 2023). At the beginning of storage, samples CM75, CM100, and CM125 showed pH values ranging from 6.25 to 6.26, consistent with findings in frankfurters containing cricket flour (Cavalheiro et al. 2023) and in beef patties with *G. assimilis* (Gomes Martins et al. 2024).

Storage time influenced the pH values of meatloaves, with lower (*p* < 0.05) values observed at the end of storage. CM0 experienced the most pronounced pH decline, whereas CM125 exhibited only a slight decrease (*p* < 0.05). Interestingly, CM75 displayed a lower pH (*p* < 0.05) than CM100 and CM125, suggesting that higher cricket flour levels may contribute to greater pH stability over time. This effect may be attributed to antioxidant and antimicrobial compounds present in cricket flour, which can inhibit the growth of spoilage microorganisms and oxidative reactions, thereby reducing metabolite production and the consequent pH variation of meat products during storage (Lone et al. 2023).

#### 3.1.5 Instrumental Color

**FIGURE 3:**
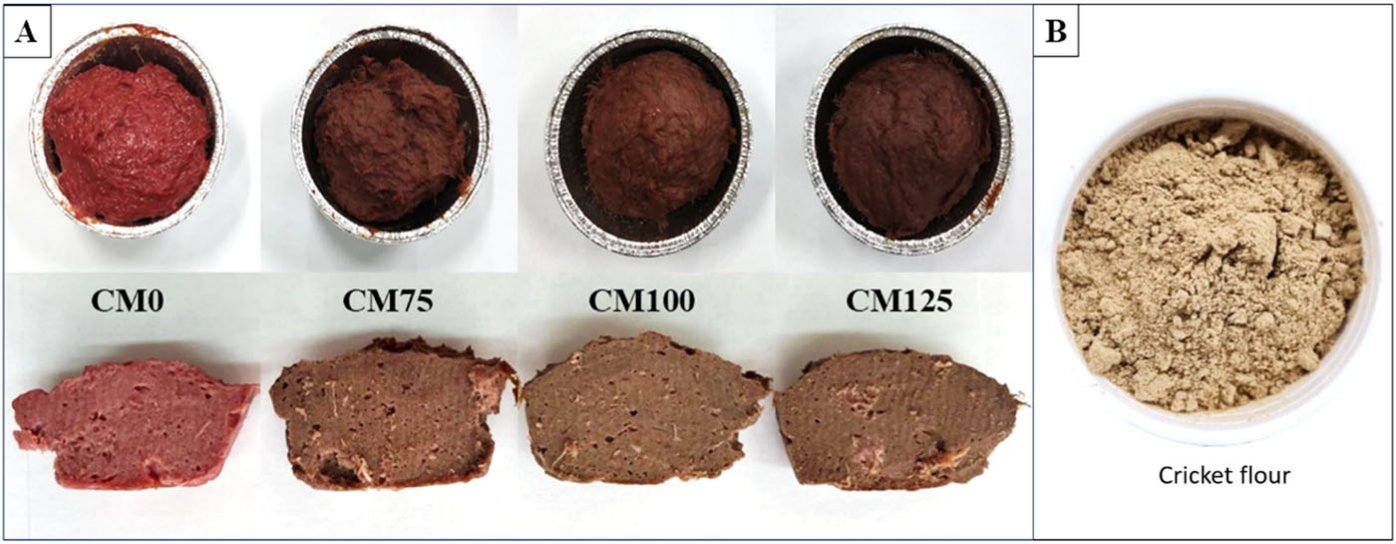
Visual observation of hybrid meatloaves (A) with different levels of cricket flour (B) as a beef replacer. CM0: meatloaf without cricket flour; CM75: meatloaf with 7.5% cricket flour; CM100: meatloaf with 10% cricket flour; CM125: meatloaf with 12.5% cricket flour.

Treatments, storage days, and their interaction significantly (*p* < 0.05) affected the color parameters (L*, a*, and b*) (Table 2). On day 0, initial L* values ranged from 55.2 to 56.4, with CM75 showing higher (*p* < 0.05) values compared to the other treatments (*p* < 0.05) (Table 2). However, the highest (*p* < 0.05) L* value was observed in the CM0 treatment at day 7 of storage (57.9). Additionally, Table 2 shows that CM0 and CM100 treatments lightened during storage by day 7, while CM125 darkened (*p* < 0.05). Meatloaves visually became darker (*p* < 0.05) as the level of cricket flour increased (Figure 3a) due to the dark brown color of the cricket flour (Figure 3b).

**TABLE 2:** Color parameters of meatloaves with different levels of cricket flour during the storage period.

Parameter	Day	Treatment				—	*P*‐value	—	—
		CM0	CM75	CM100	CM125	SEM	*t*	*T*	*t* × *T*
L*	0	55.5^C^	56.4^B^	55.4^C^	55.2^C^	0.5	***	***	***
—	7	57.9^A^	56.4^B^	56.3^B^	54.5^D^	1.3	—	—	—
—	SEM	1.3	0.2	0.5	0.3	—	—	—	—
a*	0	9.4^A^	5.4^C^	4.1^F^	4.9^D^	2.0	***	***	***
—	7	8.6^B^	5.0^D^	4.4^E^	4.2^F^	1.9	—	—	—
—	SEM	0.4	0.2	0.1	0.1	—	—	—	—
b*	0	8.3^A^	7.5^DE^	7.6^CD^	7.7^C^	0.4	***	**	***
—	7	8.1^B^	7.4^EF^	7.9^B^	7.3^F^	0.4	—	—	—
—	SEM	0.1	0.0	0.2	0.2	—	—	—	—
∆E*	0	—	4.6^C^	5.1^B^	4.5^C^	0.3	***	**	***
—	7	—	4.0^D^	5.1^B^	5.5^A^	0.6	—	—	—
—	SEM	—	0.3	0.0	0.5	—	—	—	—

*Note*: Means with different uppercase superscripts (A–F) indicate significant differences of treatment and storage day interaction. SEM: Standard error of the mean. CM0: meatloaf without cricket flour addition; CM75: meatloaf with 7.5% cricket flour; CM100: meatloaf with 10% cricket flour; CM125: meatloaf with 12.5% cricket flour. t: treatments (CM0, CM75, CM100, CM125); T: storage times (0 and 7 days); **: *p* < 0.01; ***: *p* < 0.001; n.s.: not significant.

Cricket flour addition significantly reduced (*p* < 0.05) both a* and b* values. CM0 had the highest a* values (*p <* 0.05) on days 0 and 7 of storage (9.4 and 8.6, respectively). Hybrid meatloaves showed a* values ranging from 4.1 to 5.4 at the beginning of storage. However, the storage period reduced the a* values of the CM0 treatment. Similarly, the storage period also reduced a* values on CM75 and CM125 treatments (Table 2). The lowest a* values were observed in the CM100 treatment at day 0 and in the CM125 treatment at day 7 of storage. The reduction of a* values in CM0, CM75, and CM125 during storage can be mainly attributed to the conversion of carboxymyoglobin into deoxymyoglobin and metmyoglobin (Gupta et al. 2018). Similar findings were already reported by Gomes Martins et al. (2024) in beef patties with *G. assimilis* addition being darker and less red than the control. Additionally, the highest b* values (*p* < 0.05) were observed in CM0 at days 0 and 7 of storage (Table 2). On day 7 of storage, b* values ranged from 7.3 to 8.1, with the CM125 treatment being the lowest (*p* > 0.05).

The ∆E* was calculated to evaluate the overall color difference by comparing the hybrid treatments with CM0 on days 0 and 7 of storage. Treatment, storage period, and their interaction significantly (*p* < 0.05) influenced the ∆E* values. At the beginning of storage, CM100 had the highest (*p* > 0.05) color difference related to CM0 when compared to the other hybrid treatments (Table 2). Additionally, it was possible to note that ∆E* reduced (*p* < 0.05) in CM75 on day 7 of storage, remaining stable in CM100, and increasing in CM125. The addition of cricket flour intensifies the Maillard reaction in cooked meat products, leading to the formation of pigments that interact with meat proteins, which contributes to darker initial coloration and reduced color stability during storage (Han et al. 2024). This means that the storage period had different effects in hybrid meatloaves, reducing color differences in treatments with less cricket flour addition and increasing these color differences in treatments with higher cricket flour as a meat replacer. The CM100 and CM125 treatments exhibited ∆E* values greater than 5.0, indicating noticeable color changes compared to CM0 (Bellary et al. 2016). ∆E* values higher than 5.0 were already observed in beef patties with *G. assimilis* addition (Gomes Martins et al. 2024).

#### 3.1.6 Texture Profile

The incorporation of cricket flour, storage day, and their interaction significantly (*p* < 0.05) affected the hardness and chewiness parameters of meatloaves (Table 3). Replacing lean beef meat with cricket flour increased the hardness of hybrid meatloaves (*p* < 0.05) in proportion to the level of cricket flour added. The lowest hardness values were observed in the CM0 treatment on days 0 and 7 of storage. The interaction between treatments and storage revealed distinct behaviors. Hardness decreased (*p* < 0.05) in hybrid treatments during storage, while it increased (*p* < 0.05) in CM0. Since partial replacement of meat proteins can influence the emulsifying capacity, emulsion stability, and gelling ability of cooked products through the progressive weakening of protein–protein interactions (Anzani et al. 2020), the incorporation of cricket flour led to a less rigid and more fragile protein matrix over time. Consequently, chewiness followed a similar trend to hardness, with higher (*p* < 0.05) values in hybrid treatments compared to CM0. This increase was also proportional to the cricket flour levels, with chewiness ranging from 641.69 to 790.24 (Table 3). A similar trend for hardness was also observed for chewiness, with values decreasing in treatments with higher cricket flour addition (Table 3). Similar increases in hardness and chewiness have been reported in previous studies incorporating edible insects into meat products (Kim et al. 2017; Park et al. 2017; Cavalheiro et al. 2023). These changes are likely attributable to the formation of a denser protein matrix (Youssef et al. 2011) and differences in the protein‐moisture ratio between CM0 and the hybrid formulations. However, softer texture in beef patties with *G. assimilis* was observed, probably due to the crumbly texture associated with the product (Gomes Martins et al. 2024). Springiness remained unaffected by cricket flour addition, the storage period, or their interaction (*p* > 0.05) (Table 3). In contrast, cohesiveness was higher in CM0 (*p* > 0.05) than in the hybrid treatments, potentially due to the greater swelling capacity of cricket flour (Cavalheiro et al. 2023).

**TABLE 3:** Textural parameters of meatloaves with different levels of cricket flour during storage.

Parameter	Day	Treatment				—	*P*‐value	—	—
		CM0	CM75	CM100	CM125	SEM	*t*	*T*	*t × T*
Hardness (*N*)	0	3.96^H^	7.44^D^	8.68^B^	9.98^A^	2.39	***	***	***
—	7	4.63^G^	6.59^E^	6.07^F^	8.07^C^	1.31	—	—	—
—	SEM	0.39	0.49	1.51	1.11	—	—	—	—
Chewiness (mm)	0	336.12^E^	641.69^C^	695.24^B^	790.23^A^	181.79	***	***	***
	7	390.30^D^	671.56^B^	627.89^C^	638.91^C^	119.81	—	—	—
—	SEM	31.32	19.63	39.12	87.38	—	—	—	—
Springiness (*N* × mm)	0	0.97	0.94	0.95	0.95	0.02	n.s.	n.s.	n.s.
	7	1.60	1.31	0.97	0.93	0.38	—	—	—
—	SEM	0.45	0.35	0.02	0.01	—	—	—	—
Cohesiveness	0	0.84	0.82	0.81	0.82	0.01	*	n.s.	n.s.
—	7	0.85	0.83	0.82	0.81	0.02	—	—	—
—	SEM	0.01	0.02	0.00	0.01	—	—	—	—

*Note*: Means with different uppercase superscripts (A–H) indicate significant differences of treatment and storage day interaction. SEM: Standard error of the mean. CM0: meatloaf without cricket flour addition; CM75: meatloaf with 7.5% cricket flour; CM100: meatloaf with 10% cricket flour; CM125: meatloaf with 12.5% cricket flour. t: treatments (CM0, CM75, CM100, CM125); T: storage times (0 and 7 days); **: *p* < 0.01; ***: *p* < 0.001; n.s.: not significant.

#### 3.1.7 Lipid Oxidation

**FIGURE 4:**
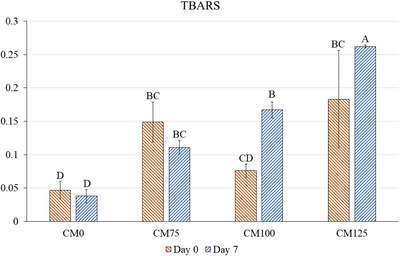
TBARS of hybrid meatloaves elaborated with cricket flour in different concentrations during storage time. Different uppercase superscripts (A–D) indicate significant differences among treatments (*p <* 0.05). Error bars correspond to the SEM. CM0: meatloaf without cricket flour; CM75: meatloaf with 7.5% cricket flour; CM100: meatloaf with 10% cricket flour; CM125: meatloaf with 12.5% cricket flour.

The addition of cricket flour, the storage period, and their interaction affected (*p* < 0.05) the TBARS values in meatloaves (Figure 4). The incorporation of cricket flour increased (*p* < 0.05) TBARS values of CM75 and CM125 treatments compared to the CM0 at the beginning of storage. The lowest TBARS values (*p <* 0.05) were observed in CM0 on days 0 and 7 of storage, while the highest was found in CM125 on day 7. No significant differences were observed for CM75 during storage; however, TBARS values for CM100 and CM125 were higher (*p* < 0.05) on day 7 than on day 0. These results were expected, since the hybrid treatments contained a higher fat content than CM0 (Table 1). In addition, the cricket flour used in this study has a predominance of unsaturated fatty acids (Cavalheiro et al. 2023), which are more susceptible to oxidation than the saturated ones. This probably contributed to the elevated TBARS values in CM100 and CM125 at the end of storage. Despite these changes, all treatments maintained TBARS values below 1.0 mg MDA/kg, the threshold commonly associated with detecting off‐flavors in meat products (McKenna et al. 2005).

### 3.2 Microbiological Properties

The microbiological counts of meatloaves during refrigerated storage are presented in Table 4. For mesophilic aerobic counts, the effects of cricket flour addition, storage period, and interaction were significant (*p* < 0.05). The addition of cricket flour increased (*p* < 0.05) the mesophilic aerobic counts of hybrid meatloaves compared to the CM0 (Table 4). The lowest (*p* < 0.05) mesophilic aerobic counts were observed in CM0 at day 0 of storage. Interestingly, a significant increase (*p <* 0.05) in mesophilic aerobic counts between days 0 and 7 was only observed in CM0. These results suggest that hybrid meatloaves may exhibit greater microbiological stability despite having higher initial counts. These findings align with previous reports of elevated initial bacterial counts in beef patties containing cricket flour (Cavalheiro et al. 2024). However, except for treatment CM75 at day 7, all the other treatments had lower than 6 log CFU/g for mesophilic aerobic counts, which is generally desired for meat products. The psychrophilic aerobic counts remained stable during storage (*p >* 0.05). As previously mentioned, the enhanced microbiological stability observed in hybrid treatments relative to CM0 during storage can be attributed to the presence of bioactive peptides with antimicrobial properties in cricket flour, which are capable of reducing the proliferation of spoilage microorganisms (Lone et al. 2023). Furthermore, no growth of *Enterobacteriaceae*, coagulase‐positive staphylococci, lactic acid bacteria, or *Salmonella* spp. was detected in any meatloaf samples throughout the storage period, confirming their good microbiological quality and compliance with safety standards.

**TABLE 4:** Microbiological properties (log CFU/g) of meatloaves elaborated with cricket flour in different concentrations during storage time.

Parameter	Day	Treatment				SEM	*P*‐value	—	—
		CM0	CM75	CM100	CM125		*t*	*T*	*t × T*
Mesophilic aerobic counts	0	2.48^C^	5.06^AB^	4.78^B^	5.01^AB^	1.25	***	***	**
	7	5.06^AB^	6.48^A^	5.15^AB^	5.35^AB^	1.09	—	—	—
—	SEM	1.67	0.86	0.82	0.63	—	—	—	—
Psychrophilic aerobic counts	0	3.06	2.63	3.48	3.10	0.40	n.s.	*	n.s.
	7	4.52	3.15	4.55	3.10	1.18	—	—	—
—	SEM	1.00	0.77	1.06	0.00	—	—	—	—

*Note*: Means with different uppercase superscripts (A–C) indicate significant differences of treatment and storage day interaction. SEM: Standard error of the mean. CM0: meatloaf without cricket flour addition; CM75: meatloaf with 7.5% cricket flour; CM100: meatloaf with 10% cricket flour; CM125: meatloaf with 12.5% cricket flour. t: treatments (CM0, CM75, CM100, CM125); T: storage times (0 and 7 days); *: *p* < 0.05; **: *p* < 0.01; ***: *p* < 0.001; n.s.: not significant.

## 4 Conclusion

Incorporating up to 12.5% cricket flour (CM125) in hybrid meatloaves significantly enhanced protein content, improved the mineral profile, and reduced processing losses without compromising texture or pH stability. Furthermore, despite its high microbial counts, the CM125 formulation exhibited good microbiological stability. However, the darker and browner color associated with higher cricket flour levels may affect consumer acceptance. To address these challenges, future research should focus on assessing hybrid meatloaves sensory properties to understand consumer preferences better. Additionally, studies exploring these products' microbiological and oxidative stability over extended storage periods would provide valuable insights into their long‐term quality and safety.

## Author Contributions

**Camila Cristina Avelar de Sousa**: investigation, writing—original draft, writing—review and editing, formal analysis, visualization. **Thiago da Matta Pires Cruz**: formal analysis, investigation. **Juliana Sant'Ana Falcão Leite**: investigation, formal analysis. **Adriel da Silva Luz**: investigation, formal analysis. **Gabriela Santana dos Anjos**: investigation, formal analysis. **Ana M. Herrero**: writing—original draft, writing—review and editing. **Maurício Costa Alves da Silva**: writing—original draft. **Claudia Ruiz‐capillas**: conceptualization, methodology, writing—original draft, resources. **Carlos Pasqualin Cavalheiro**: conceptualization, methodology, supervision, project administration, writing—original draft, funding acquisition, writing—review and editing.

## Conflicts of Interest

The authors declare no conflicts of interest.

## Data Availability

Data will be made available upon a reasonable request.
